# Takotsubo Cardiomyopathy and 5-Fluorouracil: Getting to the Heart of the Matter

**DOI:** 10.1155/2013/206765

**Published:** 2013-12-03

**Authors:** Stephanie Hui-Su Lim, Sharon Mary Wilson, Arnagretta Hunter, Jane Hill, Philip Beale

**Affiliations:** ^1^Department of Medical Oncology, Liverpool Hospital, Elizabeth Street, Liverpool, NSW 2170, Australia; ^2^Ingham Institute for Applied Medical Research, Liverpool, NSW 2170, Australia; ^3^University of New South Wales, Kensington, NSW 2033, Australia; ^4^Department of Cardiology, St George Hospital, Kogarah, NSW 2217, Australia; ^5^Department of Cardiology, Wagga Wagga Base Hospital, Wagga Wagga, NSW 2650, Australia; ^6^Riverina Cancer Care Centre, Wagga Wagga, NSW 2650, Australia; ^7^Concord Repatriation General Hospital, Concord West, NSW 2138, Australia

## Abstract

Takotsubo cardiomyopathy is a rare but increasingly recognized phenomenon, which can occur as a side-effect of chemotherapeutic agents, in particular, the antimetabolite 5-fluorouracil. We describe a case of delayed Takotsubo cardiomyopathy after 3 weeks of adjuvant 5-fluorouracil for resected rectal adenocarcinoma in a 66-year-old female, supported by angiographic, electrocardiographic, and echocardiographic features. As a complication, she developed an apical mural thrombus with subsequent cerebral thromboembolic events and was successfully anticoagulated to make a full recovery. We present a review of the literature on Takotsubo cardiomyopathy secondary to 5-fluorouracil and the rare occurrence of thromboembolic complications. As this is a significant clinical phenomenon which involves a multispeciality approach to management, oncologists and cardiologists need to recognize it as a potential toxicity of a widely administered chemotherapeutic drug.

## 1. Introduction 

Takotsubo cardiomyopathy (TCM) as a result of exposure to chemotherapeutic agents is a rare but increasing phenomenon. TCM has been noted to occur primarily in association with 5-fluorouracil (5-FU), but it has recently been described with other chemotherapeutic agents such as bevacizumab [1], cetuximab, rituximab, doxorubicin, and cyclophosphamide (R-CHOP) combination [2]. The diagnosis of TCM is primarily based on criteria from the Mayo Clinic [3], defined as transient left ventricular (LV) dysfunction extending beyond a single vascular territory, electrocardiographic changes that can mimic acute myocardial infarction, and minimal release of myocardial enzymes in the absence of obstructive coronary artery disease or acute plaque rupture. All the above criteria must occur in the absence of recent significant head trauma, intracranial bleeding, pheochromocytoma, obstructive epicardial coronary artery disease, myocarditis, or hypertrophic cardiomyopathy. We present a case of delayed TCM whilst receiving adjuvant chemotherapy for rectal adenocarcinoma.

## 2. Case Presentation

A 66-year-old Caucasian woman with no previous cardiac history or comorbidities was diagnosed with a rectal adenocarcinoma on colonoscopy performed as part of a workup for iron-deficiency anaemia in August 2010. It was less than 12 cm from the anal verge and staged as a T3N1 M0 rectal tumor on MRI and CT. She received short-course neoadjuvant radiotherapy, consisting of five fractions of 25 Gy, followed by an ultralow anterior resection and a defunctioning ileostomy, in October 2010. Histopathology revealed a poorly differentiated adenocarcinoma with infiltration into perirectal fat, with clear excision margins and 2 out of 21 lymph nodes positive for malignancy. She proceeded to commence adjuvant chemotherapy, consisting of four months of weekly 5-FU with leucovorin according to the modified Roswell Park Regimen. Dose of 5-FU was 650 mg (450 mg/m^2^) weekly with 50 mg of leucovorin. She tolerated the first two doses well.

**Figure 1:**
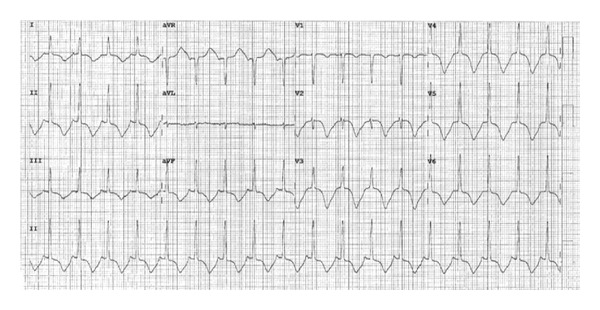
ECG on admission showing marked QT prolongation of 640 milliseconds and giant *T-*wave inversions in all leads except AVR and AVL.

She presented to the emergency department with fatigue, anorexia, nausea, and vomiting after the third week of chemotherapy, having been unwell since the third day after this dose. She described decreased oral intake, but no change in ileostomy output, and had been taking ondansetron with partial symptom relief. Blood pressure on presentation was 102/70 mm Hg with a sinus tachycardia of 135 bpm. Saturation was 100% in room air, and she was afebrile. She had a postural drop, from 100/75 mm Hg to 80/65 mm Hg on standing. Examination was otherwise unremarkable, with no signs of cardiac failure. Pathology revealed an increased creatinine of 190 *μ*mol/L (baseline 150 *μ*mol/L) with a urea of 11.5 mmol/L. She had a hypomagnesaemia of 0.3 mmol/L and a troponin I rise of 0.21 mmol/L, peaking at 0.23 mmol/L during admission. Chest X-ray revealed a normal heart size, and a renal ultrasound revealed no evidence of obstruction. ECG (Figure 1) showed marked QT prolongation of 640 milliseconds and giant *T*-wave inversions in all leads except AVR and AVL.

**Figure 2:**
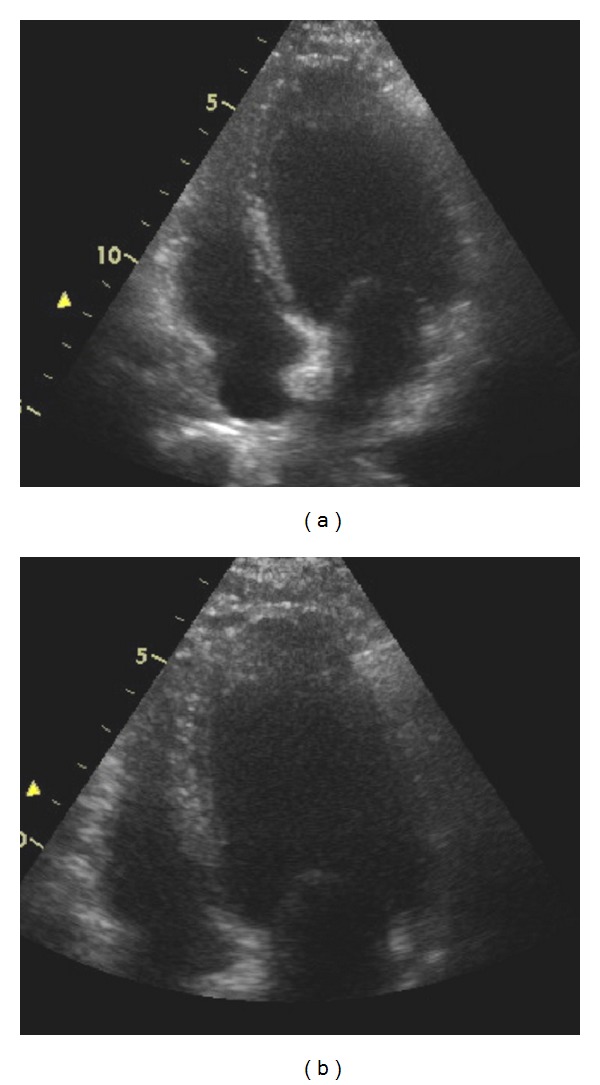
Echocardiogram showed a severely dilated left ventricular apex with associated thrombus.

The widespread QT changes on ECG were thought to be secondary to electrolyte derangement, and she received aggressive fluid and magnesium replacement. However, echocardiogram demonstrated severe systolic dysfunction with akinesis of the apex. There was preservation of the basal segments without evidence of outflow tract obstruction. Ejection fraction was calculated to be 30% at rest. A mobile echodensity was noted in the apex suggestive of a mural thrombus (Figure 2). In view of the finding of a thrombus, she was commenced on intravenous heparin. The working diagnosis was TCM with cardiogenic shock, exacerbated by dehydration.

She progressed with worsening vomiting and severe vertigo unresponsive to antiemetics. She also developed cerebellar signs, and, in view of the high likelihood of thromboembolic cerebral events, she proceeded with imaging with a brain CT which was normal. Brain MRI, however, revealed an embolic shower with a small acute infarct in the left cerebellar hemisphere with possibly two further infarcts in the mid aspect of the midbrain and high right posterior parietal parafalcine cortex.

Her symptoms improved whilst on IV heparin. Renal function normalized by day four of admission, and troponin started to trend downwards. She underwent a coronary angiogram, which revealed atheroma with no flow limiting coronary artery disease, supporting the diagnosis of TCM. Serial ECG demonstrated that the QT interval was shortening (485 milliseconds) and *T*-wave inversion was less pronounced. Repeat echocardiography demonstrated improvement of her left ventricular function to moderate segmental systolic dysfunction. After appropriate anticoagulation, her thrombus had resolved. She was transferred to rehabilitation on day 10 with no residual neurological deficits.

She did not receive further chemotherapy, and her ileostomy was reversed. Repeat ECG in March 2011 showed residual *T*-wave inversions with a normal QT interval. LV function was low-normal. Unfortunately, she developed local sacral and distant hepatic recurrence in December 2011 which was not amenable to radical curative surgery. She did receive a short course of palliative irinotecan and bevacizumab chemotherapy; however, this was ceased due to cumulative toxicities of nausea, vomiting, and fatigue. She was managed expectantly, yet she passed away in May 2013. She had not developed any further cardiac complications. Her last cardiac investigations in December 2011 showed normal ECG and low-normal LV function.

## 3. Discussion 

5-FU, a fluoropyrimidine, is used in a range of different tumor groups including gastrointestinal, breast, ovarian, and head and neck. In colorectal cancer, it was the mainstay of adjuvant treatment prior to the use of doublet chemotherapy with oxaliplatin, and it remains the gold standard of adjuvant therapy in rectal cancer. 5-FU is an antimetabolite which enters cells via facilitated uracil transport and is converted to cytotoxic nucleotides. Its mechanisms of action include inhibition of thymidylate transferase and incorporation into DNA and RNA. Common toxicities include diarrhoea, palmar-plantar erythrodysesthesia, stomatitis, and myelosuppression. Gaveau et al. first described adverse cardiac effects of 5-FU in 1969 [4], and this was again confirmed by Carpenter in 1972 [5].

High-dose infusional 5-FU was found to have a greater risk of cardiotoxicity, as reported in a prospective trial by de Forni et al. in 1992 [6]. Within the literature, up to 18% of patients are reported to develop cardiac complications after administration of 5-FU [7]. To the knowledge of the authors, there are nine previous case reports of 5-FU-induced TCM, but only two other cases occurred after multiple doses [8]. Gianni et al. [9] reported a case of TCM occurring after 5-Fluorouracil has been administered for a total of ten times. Kobayashi et al. report a case of cardiac failure occurring after 5-FU for rectal adenocarcinoma with the same regimen as that of our patient, which occurred four weeks after starting treatment [10].

TCM does not appear to be related to timing of 5-FU. Basselin et al. [11] described a 48-year-old man who developed TCM twenty-four hours after receiving the first dose of FOLFOX (5-FU in combination with oxaliplatin) chemotherapy for colon adenocarcinoma. Grunwald described a similar case in a 60-year-old woman who developed TCM twenty-six hours after the first infusion of 5-FU as part of FOLFOX [8]. Delayed response to 5-FU has previously been reported in the literature, as cardiac complications have been described up to 1 month after discontinuation of 5-FU [12]. A delayed case of TCM three months following 5-FU and cisplatin for oesophageal squamous cell carcinoma was described by Gangadhar et al., and it is believed to be the first case of chemotherapy-induced TCM in the literature [13].

The mechanism behind delayed response to 5-FU is undefined. It has been previously noted that 5-FU clearance was found to be significantly lower in women [14], and also compounding this, the active catabolites of 5-FU are reported to be involved in cardiotoxicity [15]. Whether the pharmacokinetics of 5-FU interacts with the underlying mechanism of TCM in postmenopausal women is unclear, but they may relate to similar physiological mechanisms.

Mechanisms that have been proposed include postmenopausal alteration of endothelial function in response to reduced estrogen levels that may explain the predominance in older women [16]. There have been several theories relating to sex hormones and their action on the sympathetic neurohormonal axis as well as on coronary vasoreactivity [17]. A number of features of TCM, including its association with physical or emotional stress, suggest that this disorder may be caused by diffuse catecholamine-induced microvascular spasm or dysfunction, resulting in myocardial stunning [18], or by direct catecholamine-associated myocardial toxicity.

Data on recovery from TCM is variable and has been suggested to vary from a matter of days [19] to the order of 3 to 6 months [18]. We recognize that our case was unusual due to a longer time to full recovery, but that may reflect other systemic factors which may have been influenced by the complications that developed in our patient.

Current treatment of TCM consists of supportive care and standard treatments for LV systolic dysfunction. The role of anticoagulation therapy has not yet been defined. There is inadequate literature relating to the rate of embolic events and duration of anticoagulation within the TCM patient population, but in several case reports patients were transiently anticoagulated until full resolution of LV function. The use of anticoagulation to prevent embolization in patients with known LV thrombus is supported by indirect data from observational studies in patients with LV thrombus after myocardial infarction. In these studies, anticoagulation during a period of four to six months was associated with a reduced rate of embolization [20].

Our case was complicated by development of left ventricular thrombus and subsequent embolic stroke. Kurisu et al. reported an incidence of 5.3% of apical thrombus, and within this series there was a case report of one patient undergoing a neurological event [21]. Haghi et al. reported an 8% incidence of LV thrombus, but a much lower incidence of accompanying embolic complications [22]. de Gregorio described morphological characteristics associated with increased thrombus-related embolic complications. He concluded that women >65 years of age presenting with deep/giant negative *T*-waves on admission ECG seem more likely to have thrombus-related embolic complications, which is consistent with our patient's presentation [23].

## 4. Conclusion

TCM has been increasingly noted to occur in association with 5-FU. This appears to bear no relationship to dosing interval or cumulative dose. TCM can rarely be complicated by thrombus-related embolic events, and current management has yet to be standardized; however, there is indirect data for the use of anticoagulation. As TCM is a significant clinical phenomenon which involves a multispeciality approach to management, oncologists and cardiologists need to recognize this as a potential toxicity of a widely administered chemotherapeutic drug.
